# Hypolipidemic effect and molecular mechanism of ginsenosides: a review based on oxidative stress

**DOI:** 10.3389/fphar.2023.1166898

**Published:** 2023-04-28

**Authors:** Wei Jin, Chunrun Li, Shihui Yang, Shiyi Song, Weiwei Hou, Yang Song, Quanyu Du

**Affiliations:** ^1^ Emergency Department, Hospital of Chengdu University of Traditional Chinese Medicine, Chengdu, China; ^2^ School of Clinical Medicine, Chengdu University of Traditional Chinese Medicine, Sichuan, China; ^3^ Endocrinology Department, Hospital of Chengdu University of Traditional Chinese Medicine, Chengdu, China

**Keywords:** natural medicine, ginsenosides, oxidative stress, lipid metabolism, hyperlipidemia

## Abstract

Hyperlipidemia is considered a risk factor for cardiovascular and endocrine diseases. However, effective approaches for treating this common metabolic disorder remain limited. Ginseng has traditionally been used as a natural medicine for invigorating energy or “Qi” and has been demonstrated to possess antioxidative, anti-apoptotic, and anti-inflammatory properties. A large number of studies have shown that ginsenosides, the main active ingredient of ginseng, have lipid-lowering effects. However, there remains a lack of systematic reviews detailing the molecular mechanisms by which ginsenosides reduce blood lipid levels, especially in relation to oxidative stress. For this article, research studies detailing the molecular mechanisms through which ginsenosides regulate oxidative stress and lower blood lipids in the treatment of hyperlipidemia and its related diseases (diabetes, nonalcoholic fatty liver disease, and atherosclerosis) were comprehensively reviewed. The relevant papers were search on seven literature databases. According to the studies reviewed, ginsenosides Rb1, Rb2, Rb3, Re, Rg1, Rg3, Rh2, Rh4, and F2 inhibit oxidative stress by increasing the activity of antioxidant enzymes, promoting fatty acid β-oxidation and autophagy, and regulating the intestinal flora to alleviate high blood pressure and improve the body’s lipid status. These effects are related to the regulation of various signaling pathways, such as those of PPARα, Nrf2, mitogen-activated protein kinases, SIRT3/FOXO3/SOD, and AMPK/SIRT1. These findings suggest that ginseng is a natural medicine with lipid-lowering effects.

## 1 Introduction

Changes in the modern diet of humans have led to a gradual increase in diseases related to abnormal lipid metabolism, which is represented by hyperlipidemia. This disorder is characterized by an increase in plasma total cholesterol (TC), triglyceride (TG), and low-density lipoprotein cholesterol (LDL-C) levels and a decrease in the serum high-density lipoprotein cholesterol (HDL-C) level. Hyperlipidemia is a risk factor for diseases such as nonalcoholic fatty liver disease (NAFLD), atherosclerosis, diabetes, cerebral infarction, acute myocardial infarction, and acute pancreatitis.

In the field of blood lipid control, new targets for lipid-lowering therapy have always been a hot topic of research. Methods for lowering blood lipid levels can be roughly classified into lifestyle adjustments, synthetic lipid-lowering drugs, and natural medicines. At present, statins, fibrates, niacin, and cholesterol absorption inhibitors are the most commonly used hypolipidemic drugs. However, despite their significant lipid-lowering effects, they may cause the development of liver damage, rhabdomyolysis, tumors, and diabetes. Moreover, withdrawal from these drugs may occur, limiting their clinical application to a certain extent (Ma, 2018). In recent years, it has been found that oxidative stress is closely related to elevated blood lipid levels. This has led to the clinical exploration of different antioxidative treatment strategies and the development of related drugs, such as superoxide dismutase (SOD), SOD–catalase (CAT) and glutathione peroxidase (GSH-Px) mimics, and nuclear factor erythroid-2-related factor 2 (Nrf2; encoded by gene *NFE2L2*) activators. However, researchers are yet to be able to achieve an effective concentration of these drugs in the body. Furthermore, how to suppress excessive reactive oxygen species (ROS) production from the source is the bottleneck of current research (Forman and Zhang, 2021). Traditional Chinese medicine, which includes the application of natural products as remedies, has a long history of use for the treatment of diseases related to abnormal lipid metabolism. Ginseng, the root of plants in the genus *Panax*, is a representative traditional Chinese medicine with lipid-lowering effects. Pharmacological studies have shown that this natural product has antitumor, anti-aging, and anti-fatigue effects and can regulate immunity as well as glucose and lipid metabolism (Kim et al., 2018). The main components of ginseng include saponins, polysaccharides, volatile oils, and amino acids. Ginsenosides, the major saponins of ginseng, are the key regulators of lipid metabolism. Not only do these compounds control appetite and reduce intestinal energy input by inhibiting pancreatic lipase activity, but they also inhibit lipid synthesis by activating the AMP-activated protein kinase (AMPK) pathway and stimulate energy consumption in skeletal muscle and liver tissues (Li and Ji, 2018). In animal models of hyperlipidemia, ginseng can reduce plasma levels of lipids and tissue cell concentrations of ROS and free radicals, indicating that its lipid-lowering effect is related to the inhibition of oxidative stress. Therefore, taking the inhibition of oxidative stress as a focal point, research findings on the role of ginsenosides in diseases related to abnormal lipid metabolism and the molecular mechanisms involved were systematically reviewed and summarized in this article.

## 2 Publication search strategy and selection criteria

Publications related to the review topic were searched on seven databases: PubMed, SciFinder, Scopus, The Web of Science, Embase, The Cochrane Library, and China National Knowledge Infrastructure (CNKI). Papers published from the time of establishment of these databases to December 2022 were retrieved. The keywords used were ginseng, ginsenoside, lipid metabolism disorder, hyperlipidemia, hypertriglyceridemia, hypercholesterolemia, oxidative stress, lipidemia, and substance peroxidation. There were no language restrictions on search. The following were the inclusion criteria: 1) studies using ginsenosides as intervention drugs; 2) studies with clear evaluation of lipid-lowering effects, where the blood lipid evaluation index was based on the levels of TC, TG, HDL-C, and LDL-C in serum or tissue in animal experiments and the number of lipid droplets in cell experiments; and 3) studies on oxidative stress regulation as a lipid-lowering mechanism, where the indicators of oxidative stress include the fluorescence intensity of ROS; the activities of SOD, CAT, GSH-Px, glutathione reductase (GST), heme oxygenase-1 (HO-1; encoded by gene *HMOX1*), and other antioxidant enzymes; and the content of malondialdehyde (MDA, a lipid peroxidation product). The following were the exclusion criteria: 1) literature on interventions using ginsenosides in combination with other drugs; 2) literature with unclear descriptions of research results; and 3) literature on repeated studies.

## 3 Summary of the blood lipid metabolic pathway

**FIGURE 1:**
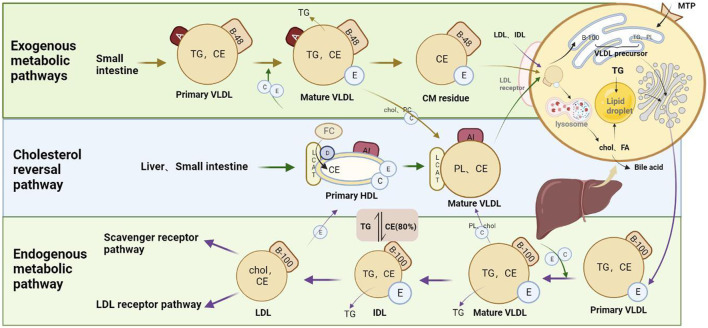
Blood lipid metabolism.

“Blood lipids” is the general name of the lipids in plasma. These include cholesterol esters, phospholipids, TGs, cholesterol, and free fatty acids (FFAs). Lipid metabolism is a complex process that involves the exogenous uptake and endogenous synthesis of lipids and the interactions of lipoproteins, receptors, and enzymes in the body. When the sources of lipids and the synthesis, metabolism, and transport of lipoproteins are disturbed, lipid metabolism disorders can develop (Zhu et al., 2016). Lipids are transported in blood as plasma lipoproteins, which can be in the form of chylomicrons (CMs), very-low-density lipoproteins (VLDLs), intermediate-density lipoproteins (IDLs), LDLs, and HDLs (Zhang and Lu, 2003). These molecules are involved in the pathways of exogenous and endogenous lipid metabolism and reverse cholesterol transport in the human body (Figure 1). Ginseng participates in the regulation of lipid metabolism mainly by interfering with enzymes, signals, and receptors in each pathway.

### 3.1 Exogenous pathways of lipid metabolism

The exogenous metabolic pathways involve the synthesis of CMs from dietary cholesterol and TGs for the purpose of carrying FFAs to the peripheral tissues for metabolism. First, dietary TGs are hydrolyzed into glycerol, fatty acids, and monoglycerides in the small intestine. Once the long-chain fatty acids and glycerol monoesters enter the intestinal epithelial cells, most of them are re-synthesized into TGs in the endoplasmic reticulum. These TGs are combined with esterified cholesterol and cell-produced apolipoproteins (apoB48, apoA) to form primary CMs, which are then released from the cells via exocytosis and enter the bloodstream through lymphatic vessels. The primary CMs combine with apolipoproteins, such as apoC-Ⅱ from HDL, to form mature CMs. When these CMs are transported to adipose tissue, skeletal muscle, the myocardium, and other tissues by blood, their apoC-II component activates lipoprotein esterase (LPL) on the surface of capillary endothelial cells, resulting in the continuous hydrolysis of TG in the core to produce fatty acids and glycerol monoesters. Of these TG hydrolysis products, 80% are absorbed by adipocytes and muscle cells and 20% are transported by albumin to the liver. Those in the liver are absorbed by hepatocytes, where they are re-esterified and used to form VLDLs, which are then secreted and transported to extrahepatic tissues. The CM remnants formed after hydrolysis of the TG in the mature CMs contain 6% apoE and 92% apoB-48. These CM remnants are ingested by hepatocytes and hydrolyzed in lysosomes, whereupon the released cholesteryl ester and fatty acids are re-esterified with hepatocyte apolipoproteins to form VLDLs.

It has been reported that ginseng may reduce the energy harvesting of rats by inhibiting pancreatic lipase activity in the exogenous pathways of lipid metabolism (Lee et al., 2013). Ginsenosides Rb1, Rb2, Rc, and Rd were found to significantly inhibit pancreatic lipase activity, which can prevent obesity by increasing fat excretion into feces (Liu et al., 2008). It has also been reported that 20(*S*)-protopanaxadiol-type ginsenoside can inhibit the expression of cholecystokinin in the hypothalamus of high-fat diet-fed mice (Kim et al., 2009). These effects indicate that supplementation with ginseng extracts can decrease obesity.

### 3.2 Endogenous pathways of lipid metabolism

The endogenous metabolic pathways involve the hepatic synthesis of VLDLs, which is transformed into the process in which IDL and LDL, LDL are metabolized by the liver or other organs. Glucose catabolism intermediates and fatty acids from both food sources and the body’s own supply are the raw materials for the synthesis of TGs in liver cells. The TGs combine with apoB100, apoE, phospholipids, and cholesterol to form new VLDLs, which are then released into the blood. These plasma VLDLs combine with HDL-transferred apoC-Ⅱ and other molecules to form mature VLDLs and IDLs successively under the action of LPL. Approximately 30%–50% of IDLs are taken up by liver cells via LDL receptors, while 50%–70% continue to circulate. The core TGs are continuously hydrolyzed by LPL so that each endogenous TG molecule is transported to extrahepatic tissues and converted into rich LDLs containing cholesteryl esters, cholesterol, and a molecule of apoB-100. After most of the LDL molecule binds to the receptor, it is engulfed by the cell. Therein, the molecule is hydrolyzed and releases free cholesterol, which can penetrate the plasma membrane of the cell and be utilized by the cell membrane. In this way, endogenous cholesterol is transported from the liver to extrahepatic tissue. The other part of the LDL molecule is oxidized (ox-LDL), and its cellular uptake is then mediated by a group of macrophage transmembrane proteins known as scavenger receptors. Under normal circumstances, macrophages will transfer the cholesterol part of LDL-C to HDL via the cell membrane protein ATP-binding box transporter A1 (ABCA1). If the transfer is impaired, the macrophages will transform into foam cells and participate in the formation of atherosclerotic plaques. Lipid-lowering statins reduce the synthesis of cholesterol in the liver by competitively inhibiting the key rate-limiting enzyme HMG-CoA reductase in the cholesterol synthesis pathway.

In the hyperlipidemic state, the levels of fatty acid transporter (FATP2, FATP5) and translocase protein (CD36) expression in hepatocytes are upregulated, which promotes TG deposition in the liver. Fatty degeneration occurs when too many fat molecules accumulate in the liver, which affects the assembly of VLDLs and inhibits the endogenous metabolic pathways of blood lipids. Recent studies (Gao et al., 2020; Yoon et al., 2021) have found that ginsenosides can reduce the hepatocyte uptake of lipids by downregulating the hepatic expression of peroxisome proliferator-activated receptor gamma (PPARγ) and its downstream target genes *CD36*, *FATP2*, *FATP5*, and fatty acid binding protein 1 (*FABP1*). Additionally, ginsenosides alleviate intracellular TG deposition by inhibiting the hepatocyte expression of sterol regulatory element binding protein 1c (*SREBP-1c*) and its target genes Fas cell surface death receptor (*FAS*) and acetyl-CoA carboxylase (*ACC*) as well as other adipogenic genes. These results show that ginsenosides can maintain normal endogenous metabolic pathways by reducing lipid accumulation in the hepatocytes and inhibiting hepatic steatosis.

### 3.3 Reverse cholesterol transport pathway

The excess cholesterol molecules that are not required for normal physiological needs have to be transported back to the liver for metabolism. This is accomplished through plasma HDLs, in a process known as reverse cholesterol transport (Zhang and Guo, 2012), which is essentially the metabolism of HDL-C. Cholesterol flows from the cells into the plasma and is converted into cholesterol ester, which in the final HDL core can eventually be transferred to TG-rich lipoproteins by the cholesterol ester transport protein (CETP), cleared in the liver by the low-density lipoprotein receptor (LDLR), or absorbed by the liver through the scavenger receptor, class B type 1 (SR-B1) pathway (Ouimet et al., 2019). In the liver, cholesterol ester is hydrolyzed, and the free cholesterol molecule is either converted into bile acid or transported to bile through ATP-binding cassette sub-family G member 5 (ABCG5) and ABCG8 for excretion in the feces. Through this mechanism, the body transfers cholesterol from the senescent cell membrane of peripheral tissues to the liver for metabolism and excretion and can prevent the accumulation of free cholesterol in the arterial wall and other tissues. Additionally, HDL also serves as the apoC reservoir, participating in the endogenous and exogenous pathways of blood lipid metabolism.

The cytochrome P450 (CYP) isoenzyme and ABCA and ABCG transporters that metabolize cholesterol are mainly regulated by PPARγ and liver X receptor-alpha/beta (LXR-α/β). In hepatocytes and macrophages, PPARγ activates LXR-α, upregulates CYP7A1 and CYP27A1, and promotes cholesterol metabolism. Studies have shown that ginseng has the effect of upregulating PPARγ expression and inducing CYP7A1, thereby regulating cholesterol metabolism.

CE: cholesterol ester, chol: cholesterol, FC: free cholesterol, MTP: microsomal triglyceride transfer protein, LCAT: cholesterol fat aminotransferase; DNL: *de novo* lipogenesis.

## 4 Hyperlipidemia and oxidative stress

### 4.1 The accumulation of free fatty acids in hyperlipidemia induces oxidative stress

Hyperlipidemia, which refers to an abnormal increase in lipid or lipoprotein levels in the blood as a result of dysregulated fat metabolism or function, is an important risk factor for cardiovascular disease (Yao et al., 2020). The clinical manifestations are high serum TC, TG, and LDL-C and low HDL-C levels. In the United States, although approximately 53% of adults have elevated LDL-C levels, less than 50% of such individuals receive treatment, and of those treated, less than 35% have adequately controlled lipid levels (Centers for Disease Control and Prevention, 2011). The deaths caused by hyperlipidemia account for nearly half of the global death toll every year (GBD, 2017 Disease and Injury Incidence and Prevalence Collaborators, 2018). The active regulation of lipid metabolism and protection of target organs are the focus of hyperlipidemia treatment.

Blood lipid levels are related to many factors, such as heredity, eating habits, race, gender, and age. In recent years, research on the pathogenesis of hyperlipidemia has mainly been carried out in the subject areas of inflammatory response, oxidative stress, endoplasmic reticulum stress, intestinal flora, and gene polymorphism (Li et al., 2019). Among such studies, many have found that the oxidative stress response plays a key role in multiple organ lesions caused by hyperlipidemia.

Oxidative stress occurs when the levels of free radicals (e.g., ROS and reactive nitrogen species) increase as a result of imbalance between the oxidative and antioxidative capacities of the body, where the decrease in antioxidative components leads to structural and functional damage in tissues and organs. An increase in serum FFAs, an intermediate product of fat metabolism, is the main cause of mitochondrial dysfunction and oxidative stress induction. Under physiological conditions, FFAs are generated from the hydrolysis of TGs under the action of a hormone-sensitive TG enzyme. These FFAs are released from the liver into the blood, where they combine with plasma albumin to form a complex that is transported to various tissues and organs throughout the body for energy utilization. Under normal physiological conditions, 1 molecule of plasma albumin can be combined with 10 molecules of FFA. However, under the state of hypertriglyceridemia, the FFAs produced by lipid metabolism greatly exceed the binding capacity of plasma albumin, resulting in a large amount of these fat molecules remaining in the blood and becoming a risk factor for oxidative stress.

For a long time, researchers have considered the mitochondrial electron transport chain (ETC) to be one of the main sources of ROS in cells and surmised that the electron carrier at the flavin mononucleotide, Fe-S cluster, and Q-binding site in complexes I and III is in a highly reduced state, making electron “leakage” possible (Schönfeld and Wojtczak, 2008; Zorov et al., 2014). On the one hand, the mobilization and utilization of FFAs decrease when the body is in the state of hyperlipidemia, and the excess fat molecules can interact directly with other components for the generation of oxygen free radicals (Loskovich et al., 2005; Schönfeld and Reiser, 2006). On the other hand, the insufficient oxidation of β fatty acids and other saturated fatty acids can destroy the mitochondrial structure. Additionally, the amphiphilic property of FFAs facilitates their integration into the inner mitochondrial membrane, thereby increasing the fluidity of the membrane structure (Chen et al., 2020). All three aspects described above are important ways through which FFAs promote electron leakage and enhance ROS generation. Moreover, some ROS molecules are generated by peroxisome and endoplasmic reticulum and by NADPH oxidase (NOX) in the cytoplasm and plasma membrane.

### 4.2 Oxidative stress is involved in hyperlipidemia-induced tissue damage

ROS is produced by enzymes (e.g., lipoxygenase) in the mitochondria, peroxisomes, and endoplasmic reticula as well as by NOX, xanthine oxidase, CYP2E1, and cyclooxygenase in the cytoplasm and plasma membrane. (Schieber and Chandel, 2014; Forrester et al., 2018). Physiological levels of ROS are involved in cellular metabolism, immune defense, proliferation, and differentiation. However, under oxidative stress conditions, the production of excess or cellular ROS shifts to that of more toxic oxidant species that can trigger pathological redox signaling and subsequent cell damage (Campbell and Colgan, 2019), thereby disrupting various signaling pathways and accelerating pathological processes by modifying proteins, promoting inflammation, inducing apoptosis, downregulating autophagy, and damaging mitochondrial function.

**FIGURE 2:**
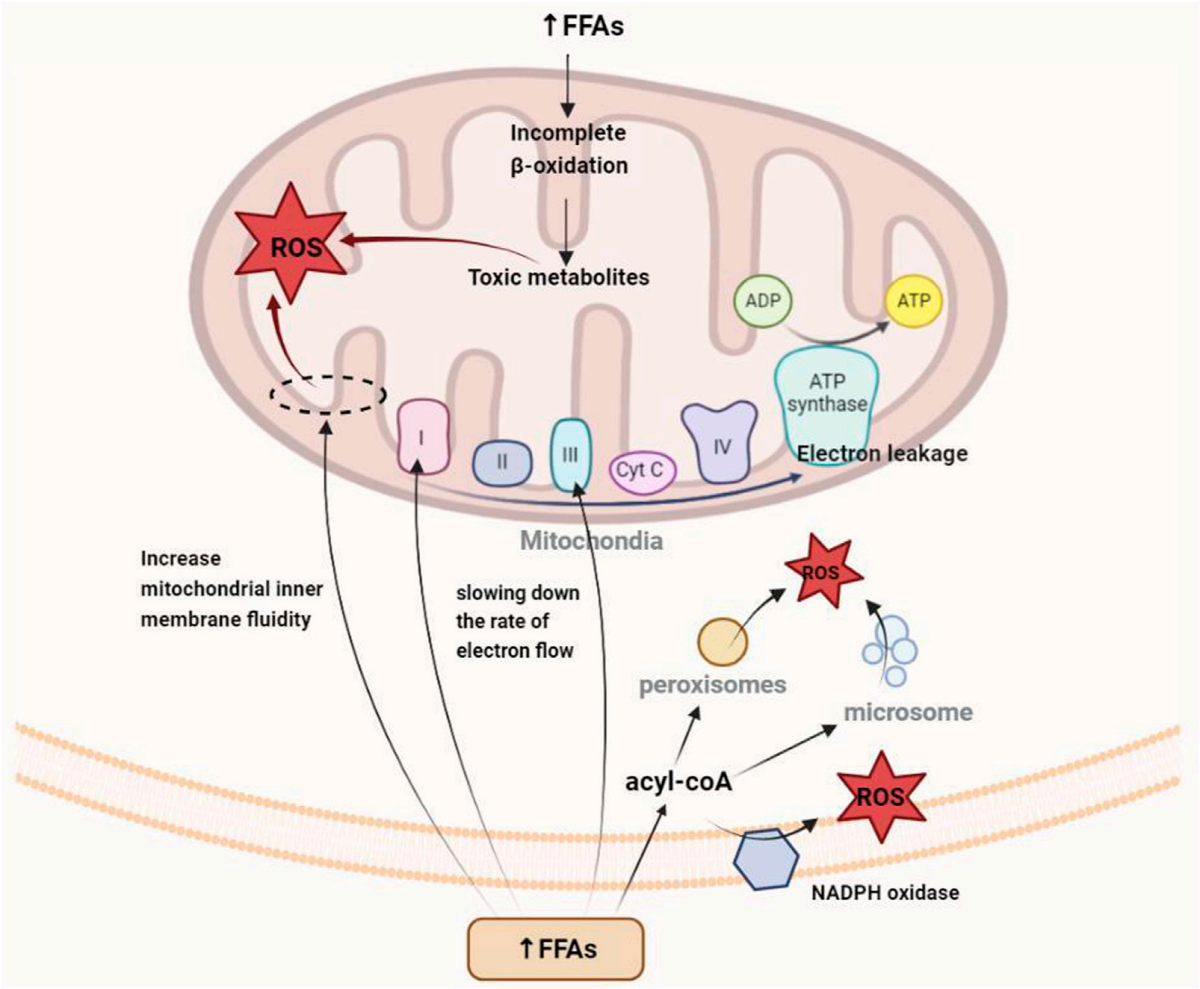
Mechanism of oxidative stress caused by the increase in free fatty acids in hyperlipidemia.

Numerous studies have shown that oxidative stress is an early event in the development of hyperlipidemia (Yang et al., 2008). The liver is the central organ of lipid metabolism (Figure 2), with the mitochondria being the key organelles responsible for the process. Oxidative stress can cause damage to mitochondrial DNA (mtDNA), phospholipids, and other molecular structures, resulting in mitochondrial dysfunction and liver damage. Additionally, apoptosis and fibrosis result in a further decline in lipid metabolism. Because of the short distance between mtDNA and the, ETC, the lack of protective histones, and the DNA repair mechanism being imperfect, mtDNA is prone to oxidative damage-induced breakage and mutation (Murphy, 2009). In addition to mtDNA and proteins, the phospholipid components of the mitochondrial membrane are particularly susceptible to ROS-induced oxidative attack, as high concentration of polyunsaturated fatty acids in mitochondrial phospholipids makes them a primary target for reaction with oxidants, resulting in the production of various byproducts of lipid peroxidation that interact with and inactivate, ETC components. Cardiolipin, a mitochondrial membrane phospholipid component that is rich in unsaturated fatty acids, carries a methylene bridge between the two double bonds of fatty acids, making it highly sensitive to ROS-induced oxidative damage (Paradies et al., 2009). Cardiolipin also exists in various mitochondrial inner membrane proteins and enzymes, including the, ETC complex and ADP/ATP transport proteins. Crystallographic studies have shown that a small number of tightly bound cardiolipin molecules exist in each crystal structure of complex III, complex IV, and ADP/ATP carriers (Maguire et al., 2017), suggesting that the phospholipid is a component of these proteins and its presence is critical for their folding, structure, and function. In mitochondria lacking cardiolipin, the respiratory supercomplex formed by complexes III and IV destabilizes (Zhang et al., 2005), and the dimer organization of the ADP/ATP vector and other supercomplexes containing it is not stable (Claypool and Koehler, 2012). Damaged cardiolipin may produce more superoxide radicals, triggering a vicious cycle of ROS-induced mitochondrial membrane damage, leading to mitochondrial dysfunction, followed by increased mitochondrial permeability due to higher pore formation and the release of cytochrome *c* as well as the promotion of apoptosis and eventual hepatocyte damage (Petrosillo et al., 2006). At the same time, oxidative stress-induced Kupffer cell activation triggers innate and adaptive immune responses, resulting in the release of proinflammatory cytokines and chemokines, and the combined effect of oxidative stress and the inflammatory response further promotes the apoptosis of hepatocytes, with fibrosis ensuing.

Additionally, oxidative stress can induce insulin resistance by impairing insulin signal transduction and causing adipokine dysregulation, leading to systemic glucose and lipid metabolism disorders. Insulin receptor (InsR) and insulin receptor substrate (IRS) are important signaling elements in the insulin signaling pathway, with the former being the initial element of insulin signaling and the latter the connection between InsR and the downstream elements of the pathway bridge. Many studies have shown that oxidative stress can interfere with the phosphorylation of InsR and IRS through multiple pathways and hinder insulin signal transduction. For example, oxidative stress can activate mitogen-activated protein kinases (MAPK) family members, such as c-Jun N-terminal kinases (JNK), extracellular regulated protein kinases (ERK), and p38MAPK, aggravating the serine/threonine residues of InsR and IRS. The degree of phosphorylation reduces the protein binding ability between InsR and IRS and the ability of the latter to activate downstream signaling molecules that contain the SH2 domain (Henriksen et al., 2011). Phosphoinositide 3-kinase (PI3K), a signaling element located downstream of IRS in the insulin signaling pathway, consists of a catalytic p110 subunit and a regulatory p85 subunit. Upon insulin stimulation, PI3K translocates to the area of IRS-1 and IRS-2 accumulation and combines with these molecules, whereupon it is activated and catalyzes the formation of a series of downstream phosphoinositides to complete insulin signal transduction. Studies have found that oxidative stress can affect the combination of PI3K and protein kinase B (AKT) by reducing the activity of the PI3K-p110 subunit, thereby affecting insulin signal transmission (Shibata et al., 2010; Wang et al., 2010). Additionally, oxidative stress can affect insulin signaling by regulating the expression of the glucose transporter type 4 (*GLUT4*) gene (Henriksen et al., 2011).

Under the continuous cycle of oxidative stress and destruction of cellular structures and functions, the body finally manifests the coexistence of hyperlipidemia, hyperinflammation, and high oxidative stress levels, leading to other serious conditions such as atherosclerosis, progressive demyelination of peripheral nerves, and sarcopenia. If left untreated, these will eventually result in multiple organ and tissue damage throughout the body.

### 4.3 Alleviation of oxidative stress helps reduce tissue damage in hyperlipidemia

To defend against oxidative damage, organisms have evolved a defense system that can be roughly divided into three lines of defense. The first one is to remove ROS, such as hydrogen peroxide. However, because oxidants react too quickly with membrane lipids, proteins, and nucleic acids to be effectively removed by exogenous small molecules, preventing the production of ROS is the focus of the first line of defense. The second line of defense involves enhancing the activity of antioxidant enzymes, and the third line is to repair or remove oxidized macromolecules (Forman and Zhang, 2021).

Inhibiting excessive oxidative stress in patients with hyperlipidemia can help toward protecting their target organs. First, clearing the overloaded FFAs is beneficial in preventing ROS overproduction. Through facilitation by the cell membrane FATPs, the FFAs produced after fat mobilization are taken up and oxidized by various organ tissues, among which the liver, heart, and skeletal muscle are the most active in this regard. In different types of tissue, members of the PPAR family regulate the transcription of genes involved in fatty acid transport and metabolism, including *FABP1*, carnitine-palmitoyl transferase 1 (*CPT1*), and PPAR-gamma coactivator 1 alpha (*PGC-1α*). Among the PPARs, PPARα is mainly responsible for the uptake of fatty acids and regulation of oxidative stress and is highly expressed in the liver (Gross et al., 2017; Liss and Finck, 2017). Studies have shown that hepatic deficiency in PPARα can impair the ability of the liver to utilize fatty acids, resulting in lipid deposition (Benhamed et al., 2016), whereas its activation can enhance the expression of fatty acid oxidation genes and reduce steatosis (Zhu and Zhao, 2017). Additionally, AMPK activation increases the rate of fatty acid oxidation by enhancing glucose uptake and oxidation in skeletal muscle cells (O’Neill et al., 2013), and this may be related to the improvement of the insulin resistance state of the body through the regulation of PGC-1α to promote the activity of the mitochondrial oxidative respiratory chain (Kukidome et al., 2006).

With regard to the existing response to excessive oxidative stress, the main strategy is to improve the activity of antioxidants in the body, including SOD, CAT, and GSH-Px. Clinical studies have shown that the levels of MDA (a lipid peroxidation product) and advanced oxidation protein product (AOPP; an oxidative stress marker) are significantly increased in patients with hyperlipidemia, whereas those of SOD and GSH-Px (free radical scavengers) are significantly decreased (Zhang et al., 2016). Nrf2 is an important regulator of the expression of antioxidant enzymes and enhancement of the endogenous antioxidative capacity (Guariguata, 2013). Studies have shown that Nrf2-dependent antioxidant genes encode almost all antioxidant enzymes, including SOD, CAT, GST, glutathione peroxidase 1 (GPX1), and HO-1. The activation of Nrf2 has been shown to reduce bisphenol A-induced lipid accumulation in the liver of mice, and its efficacy in inhibiting lipid accumulation is considered to be related to the enhanced expression of genes coding for antioxidants and detoxification enzymes (Ludtmann et al., 2014; Shimpi et al., 2017). Promoting the expression of Nrf2 and downstream SOD, HO-1, and other signals can reduce oxidative stress and improve lipid metabolism. Another important pathway that protects cells from oxidative stress is that of sirtuin 3 (SIRT3), which achieves antioxidative effects by regulating the antioxidant enzyme SOD and upregulating the activities of manganese superoxide dismutase (MnSOD) and catalase (Sundaresan et al., 2009; Qiu et al., 2010; Cheung et al., 2015). Other studies have found that complex gut microbe–microbe and microbiota–host interactions may also interfere with ROS levels and antioxidative systems at the organ level (Jones et al., 2012; Tse, 2017; Bonaz et al., 2018). However, the detailed relationship between oxidative stress and the gut microbiota remains to be elucidated.

Damaged tissue that has not been repaired or cleared in time can induce cascade reactions by releasing inflammatory factors and various cytokines. Therefore, repairing or removing oxidized macromolecules is also a key part of antioxidant therapy. Autophagy is one of the processes through which cellular components and damaged organelles are recycled. For example, damaged mitochondria are cleared through mitophagy to accommodate energy demands and support liver metabolic pathways and functions. Various mitochondrial adapter proteins, also known as mitophagy receptors, can be recruited to clear damaged mitochondria (Novak et al., 2010; Liu et al., 2012). Additionally, specific lipids such as cardiolipin are transferred from the inner to the outer membrane of damaged mitochondria where they interact with autophagosomes through lipidated microtubule-associated protein light chain 3 (LC3) for selective degradation (Kagan et al., 2016). Autophagy is related to the regulation of apoptosis. Taking the early stage of atherosclerosis as an example, autophagy can protect cells in atherosclerotic plaques from oxidative damage by degrading harmful substances, helping to inhibit apoptosis and delay aging of the vascular endothelium (Zhou et al., 2018).

**FIGURE 3:**
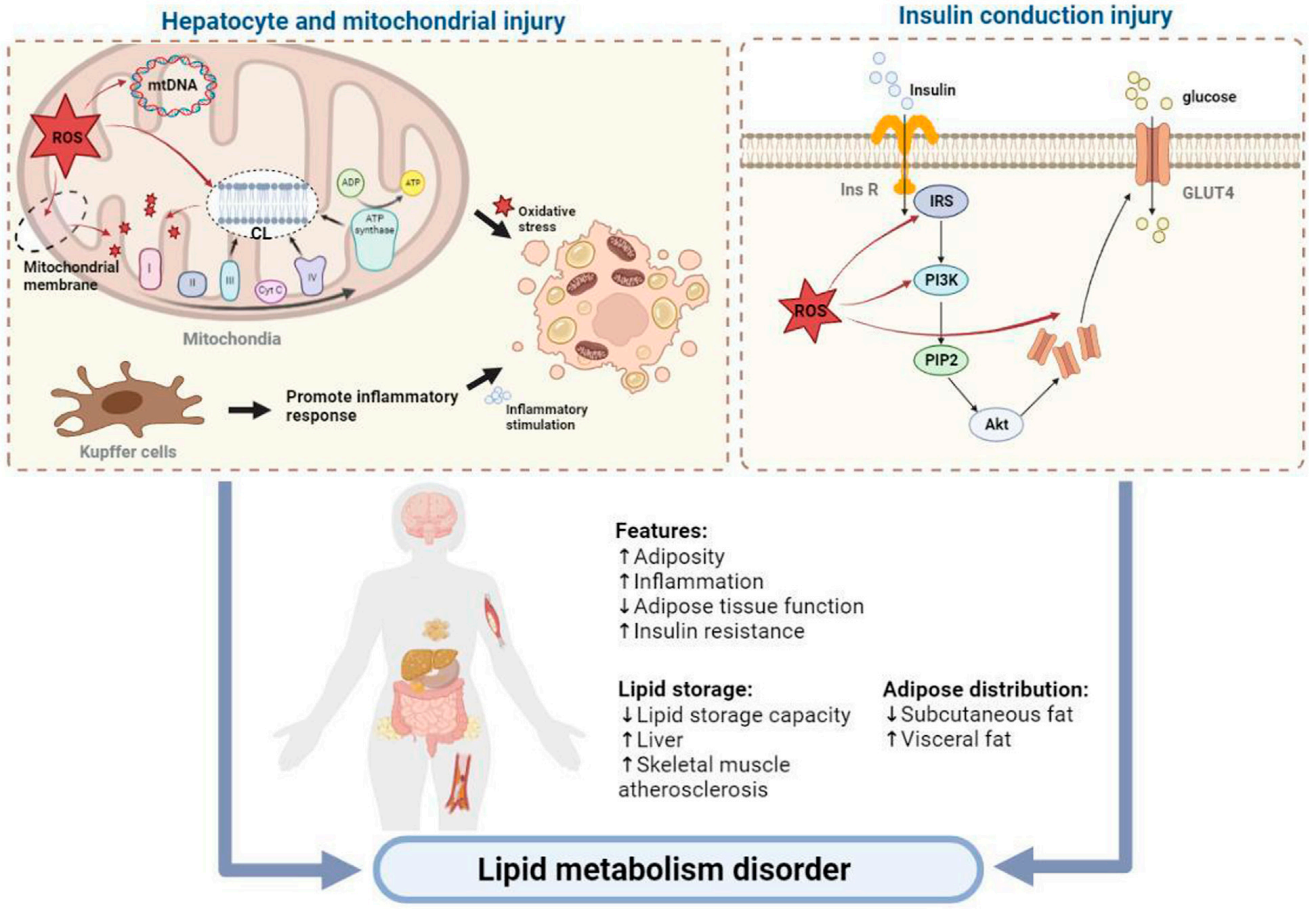
Related mechanisms of oxidative stress affecting body lipid metabolism.

In summary, hyperlipidemia can induce oxidative stress, which is the basis of tissue damage in this fat state (Figure 3). Therefore, the key to the treatment of hyperlipidemia and the prevention of secondary diseases is to prevent the production of oxidants that can cause direct damage to macromolecules, inhibit the downstream signals of oxidants that lead to inflammation or cell death signals, and increase the activity of antioxidant enzymes. Through our summarization of recent research studies, we found that ginsenosides used for the treatment of lipid metabolism disorders can exert antioxidative effects through multiple pathways and targets. These findings are expected to provide a direction for the research and development of antioxidants.

## 5 Ginsenosides regulate lipid metabolism by inhibiting oxidative stress

### 5.1 Ginseng and ginsenosides

Ginseng is one of the most widely used herbal product in the world, whether in its natural form or as a drug supplement. The plant root has a long history of use in Asian countries (especially China, South Korea, and Japan) as a dietary supplement for relieving fatigue (Shi et al., 2019). Ginseng contains ginsenosides, ginseng polysaccharides, volatile oils (terpenoids, alcohols, fatty acids, etc.), and amino acids, which have multiple biological activities, such as anticancer, antioxidative, and anti-inflammatory effects (Metwaly et al., 2019). Among them, ginsenosides are the main contributors to the known pharmacological activities of ginseng from the genus *Panax* (Seo and Kim, 2011; Lee et al., 2012). These triterpine saponins are distributed in many parts of the ginseng plant, including the berries, seeds, roots, stems, leaves, and flowers, where they have different characteristics and pharmacological effects (Attele et al., 1999; Attele et al., 1999; Podolak et al., 2010). They exist not only in Asian ginseng (*Panax ginseng*) but also in American ginseng (*Panax quinquefolius*), Chinese ginseng (*Panax notoginseng*), and jiaogulan (*Gynostemma pentaphyllum*). So far, more than 180 types of ginsenosides have been isolated from *P. ginseng* (Yu et al., 2019).

Ginsenosides are divided into three categories according to the structure of their glycosides: 20(*S*)-protopanaxadiol, 20(*S*)-protopanaxatriol, and oleanolic acid saponins. The names of ginsenosides are often abbreviated as “Rx,” where “R” refers to the root and “x” describes the chromatographic polarity in alphabetical order. Among them, Rb1 (20(*S*)-protopanaxadiol type) and Rg1 (20(*S*)-protopanaxatriol type) are the most valuable bioactive compounds. Additionally, there is a class of metabolically converted ginsenosides (derivatives), such as ginsenoside compound K (CK), which is an active metabolite of ginsenoside Rb1 and has stronger biological activity than the parent compound. Although the content of these saponins is very low, they show unique pharmacological activities.

Recent studies have found that ginsenosides play an important role in inhibiting oxidative stress, thereby preventing oxidative damage and protecting cells (Kang et al., 2013; Ong et al., 2015). Among them, ginsenosides Rb1, Rb2, Rb3, Re, Rg1, Rg3, Rh2, Rh4, F2 (Table 1), and CK have been proven to inhibit oxidative stress and alleviate hyperlipidemia by enhancing the expression of antioxidant enzymes, inhibiting the synthesis of triglycerides and cholesterol, and enhancing fatty acid oxidation.

**TABLE 1:** Ginsenosides that cause antioxidative damage.

Name	Source	Chemical constitution	Molecular weight	Oral bioavailability (OB%)	Drug similarity (DL)
Rb1	Ginseng, Panax notoginseng, Panax quinquefolium, Panax ginseng	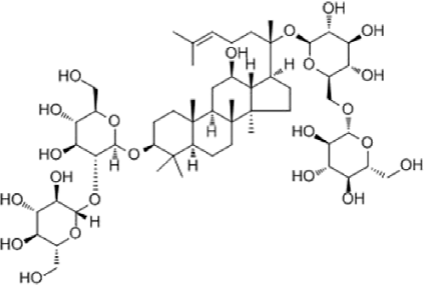	1109.295	6.24	0.04
Rb2	Ginseng, Panax notoginseng, American ginseng, gynostemma pentaphyllum	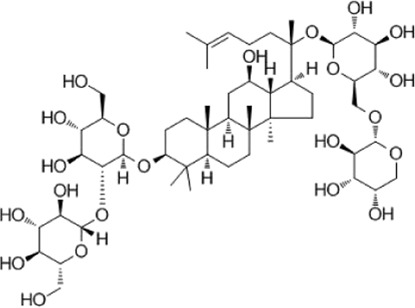	1,079.43	5.98	0.04
Rb3	Ginseng, Panax notoginseng, Panax quinquefolium, Panax ginseng	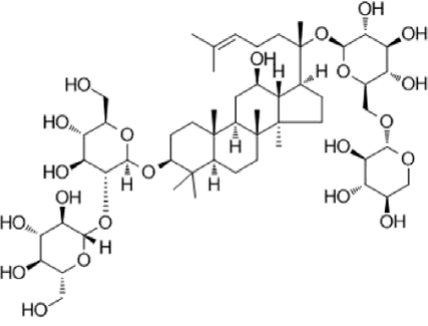	1079.269	6.02	0.04
Re	Red ginseng, gynostemma pentaphyllum, ginseng, ginseng leaf, Panax notoginseng, bamboo ginseng	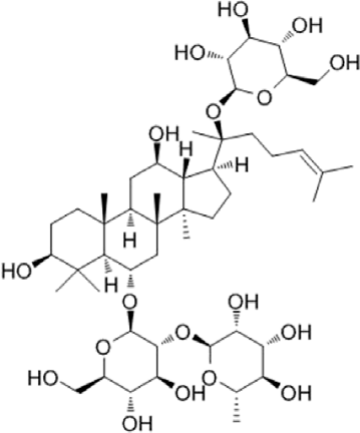	947.30	4.27	0.12
Rg1	Red ginseng, ginseng, ginseng leaf, Panax notoginseng, bamboo ginseng	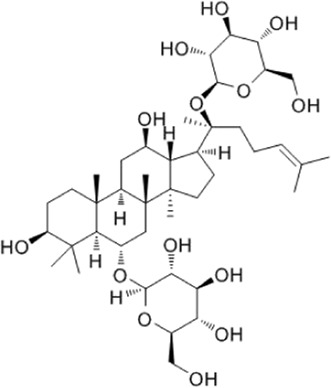	801.013	10.04	0.28
Rg3	Ginseng root, Panax notoginseng and American ginseng	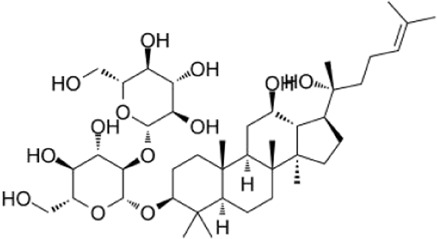	785.14	12.43	0.22
Rh2	Red ginseng, ginseng leaf, Panax notoginseng, American ginseng	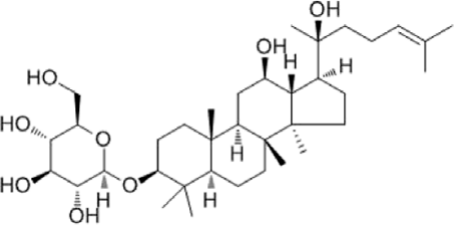	622.873	36.32	0.56
Rh4	ginseng	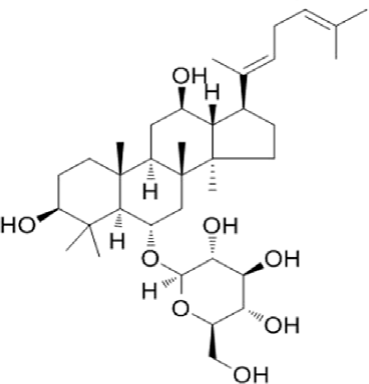	620.857	5.22	0.60
F2	Gynostemma pentaphyllum and Panax notoginseng	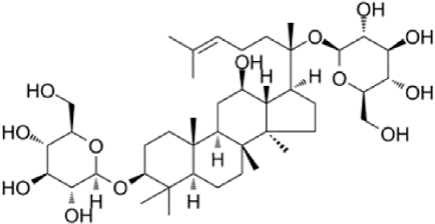	785.013	36.43	0.25

The above data is from Traditional Chinese Medicine Systems Pharmacology Database and Analysis Platform (TCMSP).

### 5.2 Ginsenosides enhance the expression of antioxidant enzymes

It has been reported that ginsenosides such as Rb1 (Zhu et al., 2022), Re (Li et al., 2013), Rg3 (Li et al., 2021), Rg1 (Xu et al., 2019), Rb3 (Wu et al., 2022), and F2 (Zhou et al., 2021) and their metabolites can improve the level of lipid metabolism through antioxidation. Under physiological conditions, Nrf2 is anchored in the cytoplasm by Kelch-like epichlorohydrin-associated protein-1 (KEAP1) to prevent its translocation to the nucleus. During oxidative stress, Nrf2 dissociates from KEAP1 and is transported to the nucleus, where it binds to antioxidant response elements, regulates the production of downstream antioxidant enzymes such as SOD and CAT, and inhibits the increase in mitochondrial ROS production (Dinkova-Kostova and Abramov, 2015; Sabouny et al., 2017). Gao et al. (2020) demonstrated that ginsenoside Rg1 could protect mice with streptozotocin-induced diabetes from inflammation and oxidative stress by activating the KEAP1/Nrf2 pathway. Ginsenoside Rg1 also protected cardiomyocytes from hypoxia/reoxygenation injury by activating Nrf2/HO-1 signaling, inhibiting JNK, and increasing the expression of SOD, GSH, and GSH-Px (Gao et al., 2020). It was found in another study that ginsenoside Rb1 could promote the nuclear translocation of Nrf2 and mediate its antioxidative effect, thereby preventing lipid peroxidation and protecting cells from oxidative stress (Li et al., 2017). *HMOX1*, one of the downstream genes of Nrf2, leads to the scavenging of free radicals accumulated by redox imbalance. SGL 121, a fabricated mixture in which the content of ginsenoside F2 has been enhanced through biotransformation, can increase the expression of HO-1 by promoting the entry of Nrf2 into the nucleus, thereby enhancing the cellular resistance to oxidative stress and helping to inhibit fat accumulation (Kim et al., 2020). Additionally, AKT and MAPK may be part of the central pathway related to Nrf2 translocation (Dai et al., 2007; Calabrese et al., 2010). Studies have found that islet dysfunction in mice with type 2 diabetes mellitus (T2DM; induced by a high-fat diet or streptozotocin) can be alleviated by ginsenoside Rh4, which it does by promoting Nrf2 nuclear translocation and upregulating the expression of HO-1, NAD(P)H dehydrogenase [quinone] 1 (NQO1), and glutamate-cysteine ligase catalytic subunit (GCLC). Conversely, an AKT signal inhibitor almost completely blocked Rh4-induced Nrf2 nuclear translocation, indicating that AKT is a key regulator of Rh4-induced Nrf2 activation. The MAPK family proteins JNK, ERK, and p38MAPK are key signal molecules in the response to mitogenic stimulation or environmental stress (Kong et al., 2001). Although it was shown that ginsenoside Rg3 could inhibit hyperlipidemia and streptozotocin-induced oxidative stress, increase the expression of SOD, CAT, and HO-1, and inhibit activation of the MAPK pathway, the exact relationship between the MAPK pathway and the antioxidant system was not further demonstrated (Li et al., 2021).

The SIRT3 pathway is also one of the most important for protecting cells from oxidative stress. It has been reported that the liver contents of TG and cholesterol in *Sirt3*-knockout mice were significantly higher than those in the wild-type animals, resulting in accelerated obesity, insulin resistance, hyperlipidemia, and steatohepatitis (Hirschey et al., 2011). Forkhead box O3 (FOXO3) is one of the downstream target proteins of SIRT3. When SIRT3 is used as an oxidative stress-related deacetylase, FOXO3 is deacetylated and captured in the nucleus, where it enhances gene transcription of the FOXO-dependent antioxidants MnSOD and catalase, acts on the ROS/Ras signaling pathway, eliminates ROS, and reduces oxidative stress damage. Many studies have confirmed that SIRT3 can protect mitochondria from oxidative damage by reducing the acetylation of FOXO3 and promoting its expression, thus alleviating oxidative stress (Zhou et al., 2021). Wang et al. (2022) used ginsenoside Rb1 to ameliorate the immune-driven liver injury induced by restraint stress and lipopolysaccharide. After 7 days, the authors found that the number of fat vacuoles in the liver tissue and the expression of MDA had decreased, whereas the activity of SOD had increased. Those researchers verified that the mechanism through which Rb1 alleviates oxidative stress is related to the upregulation of *Fox3O* gene expression.

Additionally, a HFD can change the structure of the intestinal flora and thereby its symbiotic relationship with the host, and the products of microflora also play a regulatory role in the antioxidant system of the body. Zou et al. (2022) found that ginsenoside Rb1 upregulated the abundance of the genera *Akkermansia*, *Parasutterella*, and *Bacteroides* in the intestine of HFD-fed mice; decreased the abundance of species of *Intestinimonas*, *Oscillibacter*, and *Allobaculum*; and regulated the colonic expression of FFA receptors (FFAR4, PPARγ) and genes related to intestinal barrier function (claudin 4 (*Cldn4*) encoding dense protein 4, and *Cldn2* encoding dense protein 2), antimicrobial peptides (regenerating islet-derived 3 gamma (*Reg3g*)), and oxidative stress (*SOD1*, *CAT*, and *Nfe2l2*). Rb1 also protected the intestinal barrier and downregulated the oxidative stress response, thereby regulating the structure of the intestinal flora and the profile of intestinal fatty acids. At the same time, short-chain fatty acids (SCFAs) and other beneficial metabolites produced by intestinal bacteria help to reduce ROS by affecting mitochondrial activity. For example, total saponins from the stems and leaves of *Panax ginseng* can significantly increase the levels of acetic acid, propionic acid, butyric acid, and total SCFAs by regulating the proportion of intestinal microflora, which helps to improve the activity of antioxidants such as GSH-Px and SOD (Ding et al., 2023). However, more experiments are needed to verify how intestinal microorganisms participate in the lipid-lowering effect of ginsenosides (Table 2).

**TABLE 2:** Ginseng and its active components protect organs and tissues by increasing the activity of antioxidant enzymes to reduce oxidative stress.

Drugs	Animals/Cells	Intervention results	Mechanism	References
Rb1	*In vivo*, elderly rats with fatigue syndrome after major resection of the small intestine	MDA↓, SOD↑	Activate of the PI3K/Akt/Nrf 2 pathway	Zhuang et al. (2014)
Rg3	*In vivo*, high-fat diet feeding, STZ-induced diabetic nephropathy in C57BL/6 mice	TC, TG, LDL-C↓, HDL-C↑; MDA↓, SOD, CAT↑, HO-1↑	Regulate the MAPK/NF-κB pathway	Li et al. (2021)
Rb1	*In vivo*, immune liver injury induced by restraint stress (RS) combined with lipopolysaccharide (LPS) in mice	Liver fat vacuoles↓; MDA↓, SOD↑	SIRT3/FoxO3/SOD pathway↑	Wang et al. (2022)
Rb3	*In vitro*, the lipid hepatocyte model was prepared by mixed culture medium of oleic acid (OA) and palmitic acid (PA)	Lipid stock↓, TG↓; ROS↓	the expression of Klf16, Nrf2, and Sod2↑	Wu et al. (2022)
SGL 121	*In vivo*, mice fed a high-fat, high-carbohydrate diet (HFHC); *in vitro*, HepG2 cells	TG, TC, LDL↓, HDL↑, MDA↓	Activate of AMPK, the entry of Nrf 2 into the nucleus↑, HO-1 expression↑	Kim et al. (2020)
Rb1	*In vivo*, C57BL/6J mice	TC、TG、LDL-c, adipose cell↓; Expression of the oxidative stress genes SOD 1, CAT, and NFe212 in the colon↑	Regulate the structure of the intestinal flora and the intestinal fatty acid profile	Zou et al. (2022)

### 5.3 Ginsenosides enhance fatty acid beta-oxidation

Under physiological conditions, FFAs are regulated by PPARα and its target gene acyl-CoA oxidase 1 (*ACOX1*), and the fat molecules are transported to the mitochondria via the catalytic activity of CPT1 to produce energy (Papackova and Cahova, 2015). However, when the body is in a state of high fat, the mitochondria cannot completely oxidize the excess FFAs, resulting in the production of a large number of ROS and destruction of the mitochondrial structure.

At the transcriptional level, the initiation of fatty acid oxidation in different types of cells is regulated by PPARs, of which PPARα is the key factor regulating this process and energy metabolism in the mitochondria, peroxisomes, and microsomes. As a fatty acid sensor, PPARα enhances lipid metabolism by promoting the β-oxidation of fatty acids in the peroxisomes and mitochondria, accelerating the decomposition of fatty acids, and regulating the expression of genes related to fatty acid and lipid metabolism (Wang et al., 2020). Some studies have shown that PPARα deficiency in the liver impairs the ability of the organ to utilize fatty acids, resulting in lipid deposition (Montagner et al., 2016), whereas PPARα activation enhances the expression of genes related to fatty acid oxidation and reduces steatosis (Zhu et al., 2017). It has been reported that fermented ginseng extract can increase the expression of PPARα in HepG2 cells (Chang, 2020). Rg1 has also been shown to upregulate the expression of PPARα to promote FFA oxidation and inhibit the upregulation of TG synthesis (Xu et al., 2018). Hong et al. (2018) found that the expression of PPARα was significantly decreased in mice with HFD-induced fatty liver, but Rb2 significantly improved the hepatic expression of this receptor at both the gene and protein levels, indicating that this ginsenoside could reverse the effect of HFD on PPARα expression. Similarly, using a mouse model of fatty hepatocytes, Li et al. (2021) found that the protein expression of PPARα was increased after Rb1 supplementation, indicating that this ginsenoside enhanced the oxidation of fatty acids. Furthermore, ginsenosides can upregulate *PGC-1α* gene expression (Zhao et al., 2019).

As a type of intracellular fuel receptor, AMPK also plays an important role in regulating lipid metabolism, where its activation can promote lipid catabolism by increasing the oxidation of fatty acids (Foretz et al., 2018; Fang et al., 2019). It was found that Rb1 can reduce the formation of malonyl-CoA and increase the oxidation of fatty acids by increasing the proportion of intracellular AMP/ATP, then activating AMPK phosphorylation, and subsequently inhibiting the activity of acetyl-CoA carboxylase (ACC). Rb1 can also significantly increase the expression of PGC-1α, PPAR-1α, and ACOX1, thus enhancing the oxidation of fatty acids in hepatocytes (Shen et al., 2013). In another study, ginsenoside CK was found to increase fatty acid oxidation and alleviate liver steatosis by promoting AMPK phosphorylation and upregulating the expression of PPARα and CPT1 (Hwang et al., 2018).

It has been found that the lack of hepatitis C virus core-binding protein 6 (HCBP6) can reduce the expression of CPT1A, the rate-limiting enzyme of fatty acid β-oxidation, indicating that HCBP6 deficiency aggravates the accumulation of triglycerides by inhibiting fatty acid oxidation. Additionally, HCBP6 deficiency decreases the expression of p-AMPKα. In rats fed a HFD and CCl_4_, the hepatic expression of HCBP6 decreased but was upregulated in rats treated with a mixture of water-soluble ginsenosides (Kim et al., 2011). Similarly, it was found that ginsenoside Rh2 could promote fatty acid oxidation and this effect depended on the expression of HCBP6 (Lu et al., 2020) (Table 3).

**TABLE 3:** Ginsenosides promote fatty acid oxidation, reduce oxidative stress, and protect organs and tissues.

Drugs	Model	Intervention results	Mechanism	References
Rb1	*In vitro*, fatty liver primary cells were prepared by mixed medium of oleic acid (OA) and palmitic acid (PA)	lipid droplet↓, TG↓; the fluorescence intensity of the ROS↓	the PPAR-α protein expression↑	Li et al. (2021)
Rb1	*In vivo*, HFD)-induced obese rats; *in vitro*, primary cultured rat hepatocytes	Liver weight, TG↓, lipid droplet↓	AMPK phosphorylation↑, CPT1 and ACC activities↑, PPAR-α and PGC-1α expression↑	Shen et al. (2013)
Rg1	*In vitro*, HepG2 cells were treated with 1 mmol L-1 free fatty acids for 24 h	TG, Fat droplet aggregation absorbance↓	PPARγ expression↓; the expression of PPAR-α, CPT-1, and ACOX1↑	Gao et al. (2020)
Rg1	*In vivo*, high-fat diet-induced NAFLD mice	Liver weight, TG, FFA↓; MDA↓, SOD↑	the PPAR-α expression↑	Xu et al. (2018)
Rh2	*In vivo*, high-fat diet-induced HCBP6 knockout (HCBP6-KO) in NAFLD mice	lipid deposition in the liver↓	HCBP 6 expression↑	Lu et al. (2020)
Rb2	*In vivo*, C57BL/6J mice induced by hyperfatty fatty liver fed 60% kcal for 8 weeks	Liver mass, fat vacuolar number and volume↓	the mRNA levels of CPT-1 and the PPAR-α gene and protein expression↑	Hong et al. (2018)
CK	*In vivo*, type 2 diabetic Otsuka Long-Evans Tokushima Fatty (OLETF) rats fed on a high-fat diet	lipid droplets in hepatocytes ↓, hepatic steatosis↓	AMPK phosphorylation and the expression of PPAR-α and CPT-1↑	Hwang et al. (2018)

#### 5.3.1 Ginsenosides promote autophagy and inhibit apoptosis

Autophagy is divided into three types: macroautophagy, microautophagy, and chaperone-mediated autophagy. Among them, macroautophagy is the process that has been the most studied. In autophagy, which is an important recycling process for maintaining cellular homeostasis, autophagosomes transfer aging or damaged organelles, misfolded or useless proteins, and intracellular pathogens to lysosomes for degradation (Zhang et al., 2021). For example, in pathological conditions, the mitochondria are not only the main organelles where ROS are produced but also the structures most vulnerable to ROS attack. The selective degradation of damaged mitochondria helps to restore the mitochondrial dysfunction caused by oxidative stress and reduces the accumulation of mtDNA mutations. The removal of oxidatively damaged macromolecules is beneficial to the protection of organs. Unfortunately, there are currently limited studies on the role that ginseng plays in the autophagic removal of oxidized macromolecules.

During autophagy, goblet-like structures (pro-autophagosomes) engulf cytosolic components and then close to form autophagosomes, which subsequently fuse with lysosomes, resulting in proteolytic degradation of the internalized components by lysosomal lyases. The modification of LC3 into two interconvertible forms (LC3-I and LC3-II) is required for the formation of mammalian autophagosomal membranes. First, newly synthesized LC3 in the cell is transformed into soluble LC3-I in the cytoplasm. Then, LC3-I is processed and modified by ubiquitination to membrane-bound LC3-II, which is localized in the pre-autophagosome and autophagosome. Both forms are biomarkers of autophagosomes. The LC3-II content or LC3-II/LC3-I ratio is positively correlated with the number of autophagosomes, reflecting the autophagic activity of cells to some extent. Additionally, many studies have suggested that autophagy is related to the regulation of apoptosis (Li et al., 2016), with B-cell leukemia/lymphoma 2 apoptosis regulator (BCL2) being the key protein linking and balancing the two processes to maintain homeostasis of the cellular environment. Proteins of the BCL2 family regulate the intrinsic apoptotic pathway by controlling mitochondrial outer membrane permeability. On the one hand, the phosphorylation of BCL2 at Ser70 is enhanced through interactions with BAX and BAD (BCL2-associated death promoter), inhibiting apoptosis, and on the other hand, BCL2 phosphorylation also leads to its dissociation from beclin 1 (BECN1), thereby activating autophagy (Liu et al., 2018).

**FIGURE 4:**
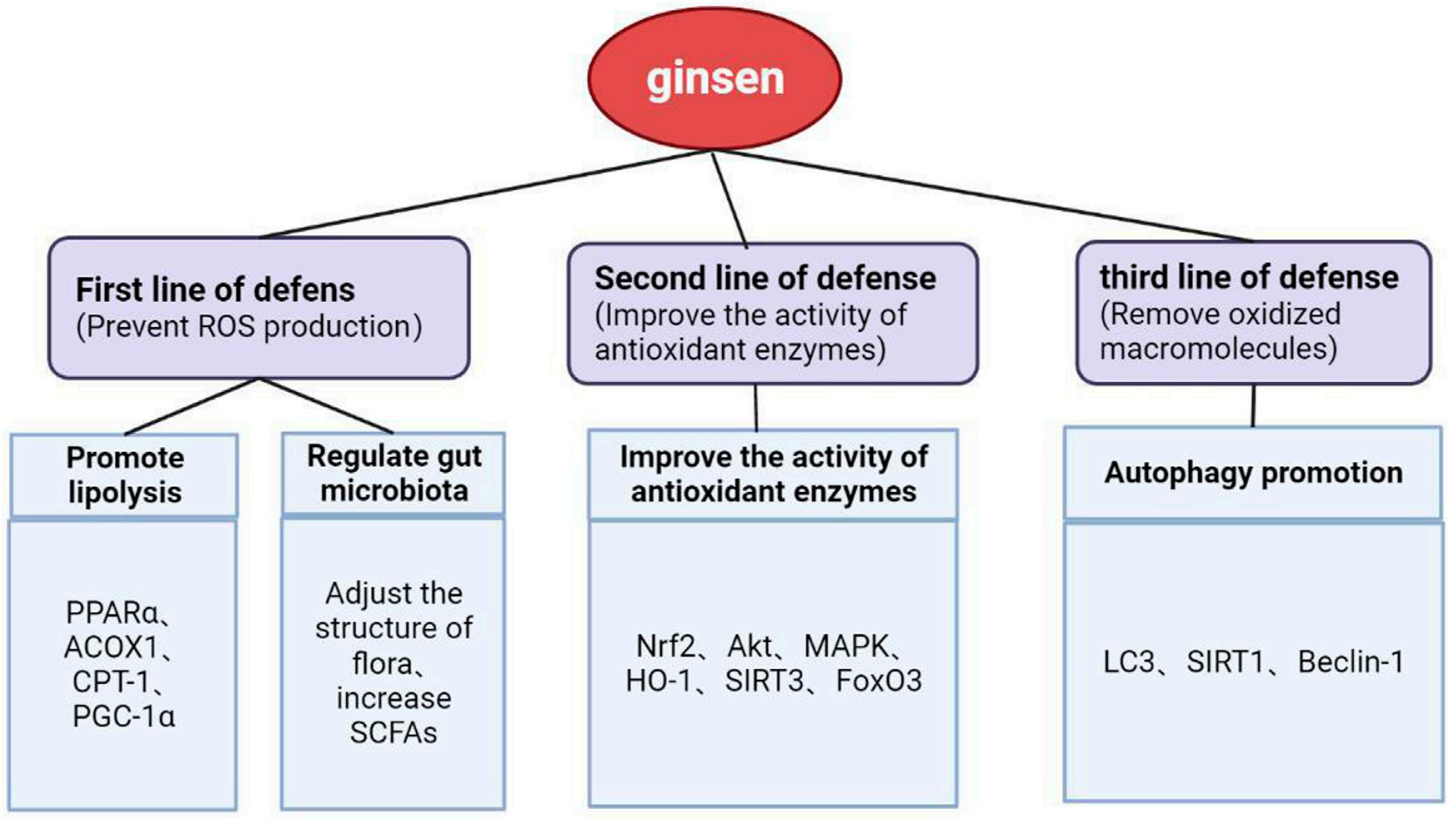
Ginseng improves lipid metabolism in hyperlipidemia by regulating oxidative stress.

Recent studies have found that the ox-LDL molecules produced during hyperlipidemia can induce vascular endothelial aging and inhibit autophagy, but ginsenosides can inhibit this process (Figure 4; Table 4). Ginsenoside Rb1 has been shown to play a protective role in mitochondrial apoptosis by regulating BCL2 family proteins (Wang et al., 2018; Zhao et al., 2018). In atherosclerosis, BCL2 accelerates autophagy by promoting the conversion of LC3-I to LC3-II to increase the autophagic flux and also inhibits apoptosis. Zhou et al. (2018) found that Rb1 feeding could reverse the levels of BCL2 and its binding protein BAX, protecting the vascular endothelium. SIRT1 was shown to prevent the progression of atherosclerosis by inhibiting foam cell formation (Kitada et al., 2016). On the basis of this study, Shi et al. (2020) further found that Rb1 could slow aging of the atherosclerotic vascular endothelium and reduce atherosclerotic plaque formation, results that were related to the increased expression of SIRT1 to reduce the autophagy induced by BECN1 acetylation. In another study, ginsenoside Rb3 was also confirmed to inhibit the ox-LDL-induced autophagic effect in endothelial cells by enhancing the expression of *BECN1* mRNA and LC3 and BECN1 proteins (Cao et al., 2018).

**TABLE 4:** Ginsenosides reduce oxidative stress by promoting autophagy.

Drugs	Model	Intervention results	Mechanism	References
Rb1	SD rats fed on a high-fat diet	Ox-LDL↓, vascular endothelial cell senescence↓	Regulate the SIRT1/Beclin-1/autophagy axis	Shi et al. (2020)
Rb3	EA.hy926 cells inoculated in DMEM/high glucose medium containing 10% fetal bovine serum and 1% double antibodies	endothelial cell damage↓	expression of Beclin1 mRNA, expression of LC3 and Beclin1 protein↑, the expression of P-mtor protein↓; autophagy↑	Cao et al. (2018)
Rb1	ApoE−/− male mice	Weight and intake↓, area of the atherosclerotic plaques↓, TC, TG, LDL-C↓, LDL-C↑	autophagy↑,apoptosis↓	Zhou et al.,2018bib_zhou_et_al_2018

## 6 Ginsenosides inhibit oxidative stress in the treatment of diseases related to abnormal lipid metabolism

### 6.1 Nonalcoholic fatty liver disease

NAFLD, the most common form of chronic liver disease worldwide, is characterized by a range of histologic features—from simple steatosis featuring fat accumulation in the liver to hepatocyte swelling, inflammation, and/or fibrosis in nonalcoholic steatohepatitis, which can lead to cirrhosis and hepatocellular carcinoma (Sayiner et al., 2016). A variety of ginsenosides can slow progression of the disease by inhibiting lipid uptake by hepatocytes, reducing intrahepatic fat accumulation, and increasing the activity of antioxidant enzymes, thereby weakening oxidative stress and protecting the hepatocytes (Table 5).

**TABLE 5:** Ginsenosides inhibit oxidative stress in the treatment of nonalcoholic fatty liver disease.

Drugs	Model	Intervention results	Mechanism	References
Rg1	*In vitro*, HepG 2 cells were treated with 1 mmol L-1 free fatty acids for 24 h	TG, the lipid droplet aggregation absorbance↓	lipid uptake↓; lipid β-oxidation↑	Gao et al. (2020)
Rg2	*In vivo*, high-fat diet-induced NAFLD mice; *in vitro*, oleic acid and palmitic acid-induced primary hepatocytes	Accumulation of lipids↓, TG, TC↓	fat synthesis↓	Cheng et al. (2020)
Rg1	*In vivo*, high-fat diet (HFD)-induced NAFLD in rats	lipid droplet, AST, ALT↓; TC, TG , LDL-C↓, LDL-C↑	β-oxidation↑	Peng et al. (2015)
Rg1	*In vivo*, high-fat diet-induced NAFLD mice	Liver weight, TG, FFA↓; MDA↓, SOD↑	fatty acid β-oxidation↑; lipid peroxidation↓	Xu et al. (2018)
Rh2	*In vivo*, high-fat diet-induced HCBP6 knockout in NAFLD mice	lipid deposition in the liver↓	fat decomposition and fatty acid oxidation↑	Lu et al. (2020)
Rb1	*In vitro*, lipid liver primary cells were prepared with a mixed medium of oleic acid (OA) and palmitic acid (PA)	the lipid droplets gather↓, TG↓; ROS fluorescence intensity↓	the oxidation of fatty acids↑; fatty acid synthesis↓	Li et al. (2021)
CK	*In vivo*, type 2 diabetic Otsuka Long-Evans Tokushima Fatty (OLETF) rats fed on a high-fat diet	lipid droplet ↓, Improvement of the hepatic steatosis	fatty acid synthesis↓; fatty acid oxidation↑	HWANG et al. (2018)
Rg1	*In vivo*, high-fat diet-induced C57/BL female mice	hepatic steatosis, FFA, TG, TC↓; MDA↓, SOD↑	antioxidant enzyme activity↑; ER stress↓	Xu et al. (2019)
Rg1	*In vivo*, SD rats, a nonalcoholic fatty liver model fed a high-glucose fatty fat diet	Liver fat vacuoles↓; GSH, SOD↑, MDA↓	oxidative stress↓; cell apoptosis in the liver↓	Xiao et al. (2019)
SGL 121	*In vivo*, mice fed a high-fat, high-carbohydrate diet (HFHC); *in vitro*, HepG2 cells	TG, TC, LDL↓, HDL↑; MDA↓	the antioxidant enzyme activity↑	Kim et al. (2020)

In the hyperlipidemic state, the increased FFA content in blood triggers the upregulation of fatty acid transporters (FATP2 and FATP5) and translocase protein (CD36) in hepatocytes, leading to the promotion of FFA absorption into the cells and thus TG deposition in the liver. When excessive fat accumulation in the liver leads to steatosis, mitochondrial function is impaired, resulting in an increase in ROS release, which further promotes the development of NAFLD. It has been found that ginsenosides can reduce lipid uptake by downregulating the expression of PPARγ and *SREBP-1c* (Cheng et al., 2020; Gao et al., 2020)*.* Additionally, for lipids that have been deposited in the liver, it has been found that ginsenosides Rg1, Rb1, Rh2, and CK can promote the expression of acyl CoA synthase 1 (CoASH1), carnitine acyltransferase 1 (CAT1), and ACOX1 in liver lipid metabolism to promote lipid oxidative decomposition and reduce liver lipid accumulation (Peng et al., 2015).

Under normal physiological conditions, oxidative stress plays an active role in “adaptive stress responses” (e.g., NAFLD detoxification, molecular damage repair, anti-inflammation, and tissue regeneration), such as by promoting cell autophagy and mitochondrial autophagy, which can effectively deal with acute exposure to cellular stressors. However, under the pathological condition, the production of excessive ROS will also promote steatosis, produce lipotoxic substances, and lead to organelle dysfunction and cell damage and death. It has been shown that oxidative stress is involved in the occurrence of NAFLD at an early stage (Mann et al., 2017). The increased ROS molecules in NAFLD can directly consume antioxidant molecules and inhibit antioxidant enzymes (Chen et al., 2020). The antioxidative capacity of hepatocytes in patients with NAFLD was shown to be significantly decreased (Reccia et al., 2017), owing to a decrease in CAT, SOD, GPX, GSH, thioredoxin, α-tocopherol, and ubiquinone levels (Koruk et al., 2004; Kumar et al., 2013; Leghi et al., 2015). Xu et al. (2019) measured the antioxidants and oxidizing compounds in a mouse model of NAFLD following intervention with different doses of ginsenoside Rg1 and metformin. The results showed that high-dose Rg1 was better than metformin in significantly reducing the MDA and FFA levels and increasing the SOD level. This indicates that Rg1 can improve antioxidation and reduce the formation of ROS and free radicals, thus ameliorating NAFLD. Xiao et al. (2019) also found that Rg1 could increase the concentrations of SOD and GSH, reduce the concentration of serum MDA, decrease oxidative stress, inhibit hepatocyte apoptosis, and alleviate the progression of the disease. These effects were related to the ginsenoside-mediated activation of AMPK and ACC phosphorylation, inhibition of the synthesis of transcription factor SREBP-1c and its target genes in adipocytes, and reduction of lipid deposition in cells (Xiao et al., 2019; Zhang et al., 2019). Additionally, it was found in liver biopsies of patients with nonalcoholic steatohepatitis that the expression of Nrf2 increased when ROS was produced at the pathological level, and drug activation of this gene in mice could reverse insulin resistance, hepatic steatosis, and liver fibrosis (Sharma et al., 2017). Moreover, the ginsenoside F2-enhanced mixture SGL121 increased the expression of HO-1 by promoting the entry of Nrf2 into the nucleus, enhanced resistance to oxidative stress, inhibited fat accumulation in the liver, and effectively eliminated liver inflammation (Kim et al., 2020). Furthermore, ginsenosides can increase the antioxidative activity of hepatocytes by upregulating the expression of antioxidant genes such as *SOD2*, *HMOX1*, and *NFE2L2* (Cheng et al., 2020).

### 6.2 Atherosclerosis

Atherosclerosis is a chronic inflammatory disease that is characterized by the accumulation of lipids and inflammatory cells in the walls of medium and large arteries. The severity of the disease is aggravated with the continuous increase in plasma cholesterol levels, especially those of LDL and VLDL, and the decrease in HDL, which are positively correlated with the incidence of the disease. Ever since lipid hydroperoxides were first found in human atherosclerotic aorta, many studies have shown an increase in lipid oxidation and other markers of oxidative stress in atherosclerotic lesions. Oxidative stress is responsible for converting LDL-C into ox-LDL, its atherogenic form. Ox-LDL plays an important role in initiating and promoting the inflammatory response and leukocyte recruitment, which inhibits or damages the defense systems (e.g., antioxidant enzymes) of the cell, thereby decreasing its ability to scavenge oxygen free radicals and finally leading to vascular endothelial cell apoptosis (Yang et al., 2017). Consequently, plaques (fatty deposits) develop on the smooth blood vessel wall, causing it to harden and damage easily. (Araujo et al., 2008). Additionally, excessive ROS can damage endothelium-dependent relaxation and trigger endothelium-dependent contraction, which are related to atherosclerotic vascular dysfunction and the subsequent development of hypertension. Studies that have tested total ginsenosides of ginseng in rats with NAFLD and ginsenoside Rg1 in mice with HFD-induced liver weight gain have shown positive effects of these saponins in decreasing the TG and FFA levels in the animals. Moreover, it was shown that ginsenoside Rh2 stimulated SOD activity and fatty acid β-oxidation (and hence increased the MDA level) *in vivo* and inhibited the formation of lipid hydroperoxides. (Nie et al., 2019) (Table 6). Ginsenoside Rg1 was shown to improve cardiac function and alleviate pathological injury of the myocardium and coronary artery in rat models of coronary heart disease. The mechanism may be related to the balance of vasomotor function and increase in the activity of antioxidant enzymes.

**TABLE 6:** Ginsenosides inhibit oxidative stress in the treatment of atherosclerosis.

Drugs	Model	Intervention results	Mechanism	References
CK	*In vivo*, a rat model of atherosclerosis prepared by high-fat diet + vitamin D3 by gavage	TC, TG, LDL-C↓, HDL-C; atherosclerotic index ↓	antioxidant enzyme activity↑	Gao et al. (2020)
Rh2	*In vivo*, a rat model of atherosclerosis prepared by high-fat diet + vitamin D3 by gavage	TC, LDL-c, AI↓; SOD↑, MDA↓	antilipid peroxidation function ↑; scavenge of oxygen free radicals	Kong et al. (2010)
Rg1	*In vivo*, a coronary atherosclerotic heart disease model rat	TG, TC, LDL-c↓, HDL-c↑; SOD, NO↑, MDA, ET↓	Balance the vasomotor function; the body’s antioxidant enzyme activity↑	Chen et al. (2020)
Rb1	*In vitro*, human aortic endothelial cells, HAECs	Cell viability, SOD, Bcl-2, HRD1↑, ROS, MDA, the rate of apoptosis, Caspase-3, Bax↓	oxidative stress↓; vascular endothelial cell apoptosis↑	Ren et al. (2022)

Ginsenosides can also protect vascular endothelial cells and stabilize atherosclerotic plaques. Rb1 may inhibit the ox-LDL-induced apoptosis of atherosclerotic endothelial cells by activating the hydroxymethyl glutaryl-coenzyme A reductase degradation protein 1 (HRD1) pathway to inhibit oxidative stress (Ren et al., 2022). Intercellular adhesion molecule 1 (ICAM-1) is a single-chain glycoprotein on the surface of vascular endothelial cells. Studies have shown that ox-LDL can upregulate ICAM-1, thus promoting leukocyte adhesion to and migration through vascular endothelial cells, whereas panaxadiol saponins can inhibit monocyte infiltration into the intima by downregulating the protein expression of aortic endothelial ICAM-1 (Li et al., 2005; Li et al., 2006; Li et al., 2009). Monocytes migrate to the intima and differentiate into macrophages, engulfing lipids to form foam cells that express scavenger receptors, which participate in the formation of atherosclerotic plaques. These plaques are extremely unstable and rupture easily, causing thrombosis. Ginsenosides Rg1, Rg3, and panaxadiol saponins have been found to inhibit the ROS-induced release of matrix metalloproteinases and degradation of the fibrous wall of atherosclerotic plaques and the basement membrane of endothelial cells, thus weakening the physical destruction caused by atherosclerotic plaques (Bao et al., 2009; Han et al., 2013).

### 6.3 Type 2 diabetes mellitus and its complications

Non-insulin-dependent T2DM, which is characterized by hyperglycemia, insulin resistance, and low-grade inflammation, is a chronic metabolic disease that affects glucose, lipid, and protein metabolism. At present, the main drugs for the treatment of T2DM are insulin, insulin secretion enhancers, insulin sensitizers, α-glucosidase inhibitors, biguanides, and glucagon-like peptide-1 receptor agonists, but these can generate adverse side effects such as hypoglycemia and gastrointestinal reactions (Jendle et al., 2016). The main pathological features of T2DM are impaired islet function and insulin resistance. Ginsenosides have been shown to improve insulin sensitivity, protect islet β cells, and promote insulin secretion, and their mechanism of action is related to antioxidation (Table 7). Therefore, ginseng has great significance as a natural medicine with low side effects and multiple targets and pathways to interfere with T2DM.

**TABLE 7:** Ginsenosides inhibit oxidative stress in the treatment of type 2 diabetes mellitus and its complications.

Drugs	Model	Intervention results	Mechanism	References
Rb1	*In vivo*, a high-glucose and high-fat diet was induced by intraperitoneal injection of streptozotocin (STZ) to construct type 2 diabetic rats	TC, TG, LDL-C↓; MDA↓, CAT, SOD, GSH-Px ↑	Improve insulin resistance and enhance the body	Zhu et al. (2022)
Re	*In vivo*,C57BL/6 mice fed on a high-fat diet	TC, TG, LDL-C↓,HDL-c↑, SOD↑, MDA↓	Protecte the antioxidant system in brain tissue and improve insulin resistance by regulating the JNK pathway	Kim et al. (2017)
Rb1	*In vivo*,High-fat feeding for 12 weeks induced obese C57BL/6J mice	Body quality↓, Blood lipid level↓, skeletal muscle endurance, and insulin sensitivity↑	Activate of proteins involve in the AMPK signaling pathway in skeletal muscle	Zhao et al. (2019)
Rg3	*In vivo*, in rats with diabetic retinopathy	Proportion of apoptotic cells, MDA and LDH expression in retinal tissues↓, SOD↑	the oxidative stress response in diabetic retinal tissue↓; activating PI3K/Akt/PKB signaling pathway; the expression of apoptosis and VE GF, ICAM-1 protein ↓	Wang et al. (2020)
Rg3	*In vivo*, C57BL/6 mice with diabetic nephropathy fed a high fat diet with a single injection of 100 mg/kg streptozotocin (STZ)	TC, TG, LDL-C↓, HDL-C↑; MDA↓、SOD, CAT↑, HO-1↑	Restrain antioxidant enzyme activity by regulating the MAPK/NF- κB pathway	Li et al. (2021)
Rb1	*In vivo*, male db/m and db/db mice	Body quality↓, CHO, LDL, Adiponectin levels↓, Lipid droplets in the liver as well as in the myocardial tissue↓; ROS, MDA↓, SOD↑	Nrf 2, GSH-Px, Sod 1, and Keap 1↑; insulin resistance and leptin resistance↓	Zhang et al. (2022)

The overproduction of ROS and elevated FFA content mediated by hyperglycemia can lead to insulin resistance by damaging insulin signaling and activating proinflammatory signaling proteins, which are related to the activation of p38 MAPK, JNK, and inhibitor of nuclear factor kappa B kinase beta (IKKβ)/nuclear factor kappa B (NF-κB) signals. Ginseng improves insulin resistance through multiple components, targets, and pathways. For example, ginsenoside Re was shown to ameliorate hyperglycemia by protecting the cholinergic and antioxidative systems in the brain of mice, thus alleviating HFD-induced insulin resistance (Kim et al., 2017). Chen et al. (2016) pointed out that ginsenosides Rb1 and CK could improve the IRS-1/PI3K/AKT insulin signaling pathway in adipose tissue by inhibiting the activation of NOD-like receptor protein 3 (NLRP3) inflammasomes and thereby improve insulin resistance. IRS-1 is the main substrate of insulin resistance. It was found that ginsenoside Re could inhibit JNK, MAPK, and serine phosphorylation of IRS-1 in 3T3-L1 adipocytes and HFD-fed rats and reduce insulin resistance (Zhang et al., 2008). Skeletal muscle cells, in which metabolism is mainly regulated by AMPK, are the main targets of insulin. Ginsenoside Rb1 can significantly reduce serum FFA levels and increase insulin sensitivity by activating the AMPK signaling pathway (Zhao et al., 2019).

Additionally, the antioxidative effect of ginsenosides can protect islet cells. Previous studies have shown that ginsenoside Rb1 can protect pancreatic β cells by inhibiting the apoptosis and oxidative stress induced by hyperglycemia and selectively reduce hydroxyl radicals and hypochlorite, the two strongest ROS (Chen et al., 2012; Lü et al., 2012). Ginseng berry extract (mainly ginsenoside Re) administered intragastrically (100 and 200 mg/kg) to mice with streptozotocin-induced T2DM for 10 weeks could promote β-cell proliferation and insulin secretion and decrease the blood glucose level in the animals (Park et al., 2012).

As mentioned above, oxidative stress is related to the pathogenesis and progression of diabetic vascular complications, such as retinopathy, nephropathy, and cardiovascular disease. Hyperglycemia can increase the level of ROS in endothelial cells through polyols and the advanced glycation end products and their receptors (AGEs/RAGEs), then activate NF-κB, and subsequently induce vascular endothelial inflammation and thrombosis by enhancing the expression of various related genes, including those coding for vascular endothelial growth factor (VEGF), vascular cell adhesion molecule-1 (VCAM-1), and endothelin-1 (ET-1). ICAM-1 and VCAM-1 are the main biomarkers of endothelial cell injury. One study found that ginsenoside Rg3 could inhibit apoptosis and VEGF and ICAM-1 protein expression by reducing oxidative stress in the diabetic retina and activating the PI3K/AKT signaling pathway (Wang et al., 2020). Ginsenosides can also upregulate the expression of vascular endothelial antioxidant enzymes (e.g., Nrf2 and NQO1), thereby playing a therapeutic role in diabetic nephropathy (Wu and Wang, 2021). Similarly, they upregulate the expression of genes encoding Nrf2, GSH-Px, SOD1, and KEAP1 to reduce oxidative stress and thereby improve the tissue pathological changes of diabetic cardiomyopathy (Zhang et al., 2022).

## 7 Discussion

According to current research on the pathogenesis of hyperlipidemia, the pathogenic factors are highly complex. This not only hampers the research and development of therapeutic drugs but also encourages the further study of the role that natural products can play in the regulation of lipid metabolism. Ginseng is widely used to treat various diseases in Asian countries, and the hypolipidemic and antioxidative effects of the plant roots have been verified. Ginseng contains saponins, sugars, volatile components, organic acids and their esters, proteins, enzymes, sterols and their glycosides, peptides, nitrogen compounds, lignins, flavonoids, vitamins, inorganic elements, and other components. The main active components are ginseng polysaccharides and ginsenosides, with the latter being the most abundant (Gao and Lu, 2021). At present, more than 180 ginsenosides have been isolated from *Panax ginseng*, among which ginsenosides Rb1, Rb2, Rb3, Rg3, Rh2, Re, Rg1, Rh4, and F2 have demonstrated effects in the treatment of hyperlipidemia.

**FIGURE 5:**
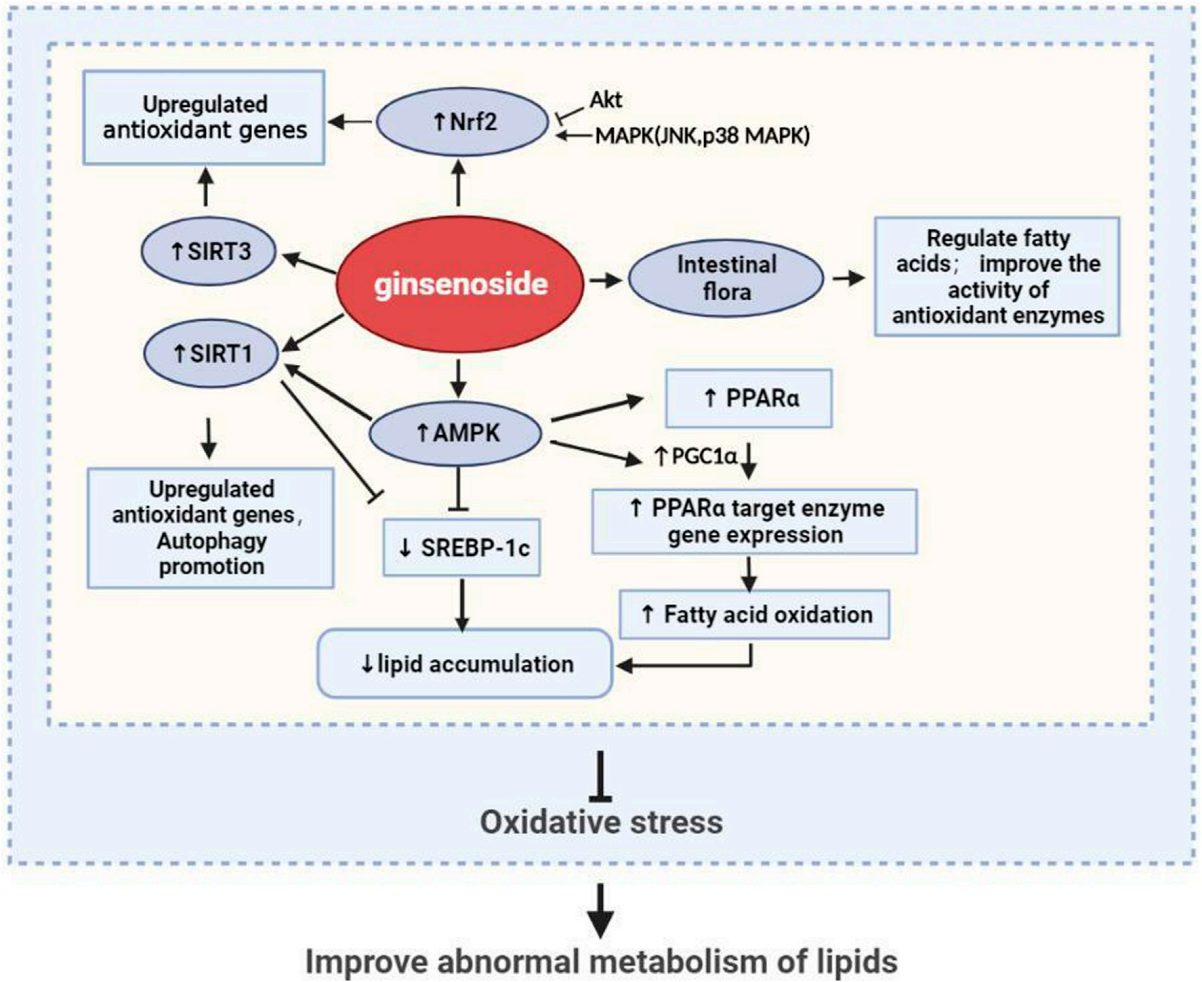
Ginseng and its active ingredients reduce lipid accumulation and promote antioxidation through multiple pathways.

In general, ginsenosides have been proven to treat hyperlipidemia and its secondary diseases by inhibiting oxidative stress *in vitro* and *in vivo* through their prevention of ROS production, enhancement of antioxidant enzyme activity, and removal of oxidative macromolecules, using processes that involve PPARα, Nrf2, MAPK, SIRT3/FOXO3/SOD, AMPK/SIRT1, and other signaling pathways (Figure 5). Nrf2 and AMPK are the most-studied signaling factors at present. Among the various ginsenosides, Rg1 and Rb1 are the most promising adjuvant drugs for the treatment of hyperlipidemia.

Although the effect of ginsenosides in improving lipid metabolism in hyperlipidemia by inhibiting oxidative stress has been investigated in many preclinical studies, there are still not many clinical trials of these compounds. Therefore, further high-quality studies are needed to determine the clinical efficacy of ginsenosides. Moreover, some studies have only shown that ginseng affects the relevant markers of oxidative stress and the indexes of lipid metabolism in serum and tissue cells. Although the data obtained from the measurement of these markers clearly show that oxidative stress is closely related to lipid metabolic disorders such as fatty liver and atherosclerotic diabetes, the biomarkers provide limited information about the type, number, and location of ROS as well as their participation in specific pathophysiological processes. The causal relationship between oxidative stress and lipid metabolism disorder in hyperlipidemia needs to be demonstrated using a more rigorous experimental design. The findings summarized in this review suggest that hyperlipidemia causes excessive FFA flow into the liver, resulting in electron leakage of the liver mitochondrial, ETC. Subsequently, oxidative stress is caused by ROS accumulation, resulting in oxidative stress damage to the mitochondria and other cell structures, which further inhibits lipid metabolism, forming a vicious cycle of hyperlipidemia and oxidative stress. However, mitochondria are not the only source of ROS. The other sources of excess ROS in lipid metabolism disorders remain to be identified and the related pathogenesis needs to be further explored.

## 8 Conclusion

At present, the treatment of hyperlipidemia and prevention of its secondary lesions remain a challenge to healthcare providers. Ginseng, as a famous natural Chinese herbal medicine with effective bioactive components (especially ginsenosides), has proven to be capable of lowering lipid levels through various mechanisms. In recent years, there has been vast progress made in research on the positive effects of ginseng in alleviating antioxidative stress and improving lipid metabolism. In this review, we have comprehensively summarized recent studies on the effects of ginsenosides on lipid metabolism disorders in animal models of hyperlipidemia and secondary diseases, such as diabetes, NAFLD, and atherosclerosis. The current findings show that ginseng can inhibit oxidative stress and improve the level of lipid metabolism in hyperlipidemia by strengthening the expression of antioxidant enzymes, promoting fatty acid β-oxidation, and promoting autophagy. Its molecular mechanism is also related to the PPARα, Nrf2, MAPK, SIRT3/FOXO3/SOD, AMPK/SIRT1, and other signaling pathways. In conclusion, current pharmacological research appears to support the hypothesis that ginsenosides regulate lipid metabolism by regulating oxidative stress. However, the definitive relationship between oxidative stress and hyperlipidemia still needs to be verified through further studies, the results of which would have great significance for the research and development of new drugs for the treatment of hyperlipidemia, especially the use of ginseng and its ginsenosides.
